# CaMoDi: a new method for cancer module discovery

**DOI:** 10.1186/1471-2164-15-S10-S8

**Published:** 2014-12-12

**Authors:** Alexandros Manolakos, Idoia Ochoa, Kartik Venkat, Andrea J Goldsmith, Olivier Gevaert

**Affiliations:** 1 Department of Electrical Engineering, Stanford University, CA, USA; 2 Biomedical Informatics Research/Department of Medicine, Stanford University, CA, USA

## Abstract

**Background:**

Identification of genomic patterns in tumors is an important problem, which would enable the community to understand and extend effective therapies across the current tissue-based tumor boundaries. With this in mind, in this work we develop a robust and fast algorithm to discover cancer driver genes using an unsupervised clustering of similarly expressed genes across cancer patients. Specifically, we introduce CaMoDi, a new method for module discovery which demonstrates superior performance across a number of computational and statistical metrics.

**Results:**

The proposed algorithm CaMoDi demonstrates effective statistical performance compared to the state of the art, and is algorithmically simple and scalable - which makes it suitable for tissue-independent genomic characterization of individual tumors as well as groups of tumors. We perform an extensive comparative study between CaMoDi and two previously developed methods (CONEXIC and AMARETTO), across 11 individual tumors and 8 combinations of tumors from The Cancer Genome Atlas. We demonstrate that CaMoDi is able to discover modules with better average consistency and homogeneity, with similar or better adjusted *R*^2 ^performance compared to CONEXIC and AMARETTO.

**Conclusions:**

We present a novel method for Cancer Module Discovery, CaMoDi, and demonstrate through extensive simulations on the TCGA Pan-Cancer dataset that it achieves comparable or better performance than that of CONEXIC and AMARETTO, while achieving an order-of-magnitude improvement in computational run time compared to the other methods.

## Introduction

Traditionally, medical science has converged upon cancer treatment strategies specific for each type of tumor (organized by the affected tissue), such as breast cancer, lung cancer, etc. Recently, however, there has been a significant effort by the research community to mine and discover shared molecular similarities across different tumors. For example, a recent study by The Cancer Genome Altas (TCGA) [1] has shown that basal-like breast cancer has more similarities, genomically speaking, to high-grade serous ovarian cancer than to other subtypes of breast cancer.

The statistical evidence for molecular, proteomic and epigenetic signatures within and across various tumors is fundamentally interesting, both from the perspective of scientific discovery and its potential impact on personalized medicine. For instance, discovering such similarities at various molecular levels can suggest an unified clinical treatment strategy to combat tumors in different anatomic sites.

Central to our discussion is the knowledge that a small number of important genes, known as "regulatory" or "driver" genes, play a crucial role at the molecular pathway level and directly influence the expression of several other genes. This network of genes, where these driver genes are connected with other downstream targets, is often modeled as a module network [2]. It seems natural that some of these regulatory genes should be able to explain the variability of gene expression in genes that appear downstream in these biological pathways. Thus, researchers are attempting to identify the module network structure based on gene expression data in cancer patients, using machine learning techniques. For example, in [3], the authors identify the module network structure in ovarian cancer. Until now, research efforts have mainly focused on studying and analyzing tissue dependent genomic patterns. TCGA [4] has collected and analyzed a large amount of data from different human tumors to discover molecular aberrations at the DNA, RNA, protein and epigenetic levels. Recently, the Pan-Cancer initiative has been created to compare the first 12 tumor types profiled by TCGA. In the era of modern medicine and big data, there is an additional need to connect the dots across different cancers, which poses a computational challenge of its own given the large volumes of patient data. This motivates the requirement of a scalable solution to the problem of module discovery in cancer. Motivated by the aforementioned reasons, we are interested in investigating both intratumor and inter-tumor genomic similarities by using the Pan-Cancer TCGA data for our study, with a focus on robustness and scalability. As a step towards solving this important problem, we present **CaMoDi**.

CaMoDi is a novel algorithm for Cancer Module Discovery, which discovers the latent module structure for a given gene expression dataset. Several methods have been previously proposed in the literature for this purpose, such as CONEXIC [5] and AMARETTO [3]. CaMoDi displays multiple advantages over previously proposed methods. These include its speed, scalability with the size of the data (both in the number of genes and the number of patients), as well as its reliability in discovering consistent clusters of genes across different train-test bootstraps of the cancer data. These characteristics make the algorithm suitable for discovering modules within and across tumors of different types.

We perform an extensive comparative simulation study between CaMoDi, CONEXIC, and AMARETTO over 11 tumors of the Pan-Cancer data set, and over 8 different combinations of tumors. To our knowledge, this is the first systematic appraisal of module discovery algorithms across a variety of tumors. Our study shows that CaMoDi is competitive with the other two algorithms, and is in many cases significantly better on a host of performance parameters that we describe below. Further, CaMoDi is able to discover modules in a timeframe that is an order of magnitude smaller than the other two methods. This has important implications for applications of CaMoDi not possible with the other algorithms. For instance, the current implementation of CONEXIC leads to excessively high run times in module discovery across combinations of several different tumors from the PanCancer data. On the other hand, as is demonstrated in our results, CaMoDi is able to discover robust modules of high quality across several tumors in very short run times.

The rest of this paper is organized as follows. In Section we review the previously proposed algorithms for module identification and introduce the proposed algorithm CaMoDi. We also describe the format and type of data used in this study, and discuss the performance evaluation criteria in detail. In Section we present the comparison results. In Section we discuss the findings of our study. Finally, we present concluding remarks in Section.

## Materials and methods

We formulate the module discovery problem as an unsupervised clustering problem in the gene space. In other words, we seek to perform an unsupervised clustering of the genes so that genes in each cluster are roughly expressible in terms of a small number of regulatory genes. This is known as a module-based approach to represent genomic profiles of tumors.

In this section, we introduce CaMoDi. We describe our method in detail, and compare it with two state-of-the-art techniques in the domain of module discovery, AMARETTO [3] and CONEXIC [5]. We provide a brief description of these two procedures, which will act as benchmarks for comparison, and refer the reader to the associated references for further details.

### Algorithms

As outlined above, the goal of these methods is to search for genes whose expression across samples (patients) can be explained well by a small number of regulatory genes. Even within this framework, there is an important difference between CONEXIC and the other two methods (AMARETTO and CaMoDi). While the latter two algorithms cluster together genes whose expression can be explained as a sparse linear combination of regulatory genes, CONEXIC considers a probabilistic model in which each cluster is represented by a regression tree, where each node is in turn associated to one of the regulators that belongs to the cluster.

#### AMARETTO

**AMARETTO **is an iterative clustering algorithm originally proposed in [3], where it was applied to dissect molecular profiles of ovarian cancer. AMARETTO finds gene clusters whose centroid is well approximated by a sparse linear combination of the regulatory genes, i.e., the center of a cluster is expressible in terms of a few regulators. We here provide a brief overview of the method and refer the reader to [3] for details:

*K−***means clustering step**: the genes are clustered into groups using standard *K−*means with *K *clusters (modules).

**Sparsification step**: Then, the centroid of each cluster is expressed in terms of the regulatory genes using linear regression with *L*_1_ and *L*_2_ regularization. This is also known as elastic net regularization [6]. After this step, each module (cluster) contains a set of genes whose average expression is described using a small number of cancer driver genes. Finally, the correlation coefficient of each gene with the sparse representation of all the centroids is calculated.

**Gene re-assignment step**: Each gene is re-assigned to the cluster whose centroid it is most positively correlated with. The algorithm repeats the *K−*means clustering and sparsification steps until the gene reassignment process converges based on less than 1% of the genes being reassigned or a maximum number of iterations being reached.

It should be noted that in [3], AMARETTO was presented as a method that integrates the copy number and DNA methylation data. However, in the current and latest implementation of AMARETTO, only the gene expression data is used, which is also the case for CaMoDi. This allows for an objective comparison of the two algorithms.

#### CaMoDi

We now present the main focus of this work **CaMoDi**, a novel approach towards fast cancer module discovery. The main purpose of the algorithm is identical to that of AMARETTO, and other procedures for module discovery, i.e., it seeks to find combinations of genes whose expression can be explained as a combination of regulatory genes. Specifically, CaMoDi attempts to create clusters of genes whose expression can be explained through sparse linear combinations of the expression of regulatory genes. The four steps of the proposed algorithm are described below. Details of the parameters used in CaMoDi appear in the Additional File 1.

**Gene sparsification step**: Each individual gene is expressed in terms of a few regulatory genes via elasticnet regression [6] with a specified maximum number of regulators. Specifically, the *L*_2 _regularization and the maximum number of regulators, denoted as *C*_1_, are user-specified parameters. Thus, via elastic-net regression, we express each gene as a linear combination of {1, 2, . . . *, C*_1_} regulatory genes. That is, every gene is mapped to *C*_1 _vectors in which the first vector has only one non-zero value, the second has two non-zero values, and so on, i.e., the expression of each gene is approximated as a weighted sum of the expression of one, two, and up to *C*_1 _regulators. We call the vector that contains *p *non-zero values (i.e., only *p *regulators are used to describe a gene), a *p−sparse *representation of this gene.

*K−***means clustering step**: A standard *K−*means clustering of the *S*_1_*−sparse *representations of all the genes is performed, where *S*_1 _is a parameter provided by the user, referred to as the *initial sparsity*. We calculate the centroids of each cluster as the average of the *S*_1_*−sparse *representations of the genes that belong in said cluster.

**Centroid sparsification step**: The centroid of each cluster is expressed in terms of the regulatory genes using elastic-net regression. In particular, the user specifies the *L*_2 _regularization and the maximum number of regulators to explain the centroids' expression, denoted as *C*_2_. The final *p−sparse *representation of each centroid is cross-validated in the following way: the average expression of all the genes that belong to the cluster (by using the initial gene expressions and *not *their *S*_1_*−*sparse representation) is computed, and the representation of the centroid which gives the highest average *R*^2 ^using a 10*−*fold cross validation over the genes of the cluster is found. This is then used to rank the clusters by their *R*^2 ^performance across all the genes affiliated with those clusters.

**Cluster filtering step**: In this step the best *P *% of the clusters are retained. Alternatively, CaMoDi also retains those clusters that exhibit an *R*^2 ^greater than *R_thresh _*and contain between *N_min _*and *N_max _*genes. Finally, the algorithm repeats the Gene Sparsification, *K−*means Clustering and Centroid Sparsification steps on the genes contained in the remaining clusters after incrementing *S*_1 _by 2.

In summary, first CaMoDi identifies possible sparse representations of each gene expression as a linear combination of different number of regulators. Second, it clusters the genes using only their *S*_1_*−sparse *representation, and identifies if the clustering leads to any module of high quality (quantified through the *R*^2 ^metric calculated using the initial gene expressions). Finally, it discards the remaining clusters, and decreases the sparsity (i.e., increases *S*_1 _in the *S*_1_*− *sparse representation of each gene) for the remaining genes, and performs another clustering. In each step it keeps at least *P *% of the clusters. In summary, CaMoDi tries to find good clusters of genes which are expressed with the same number of regulators, starting from clusters which need few regulators and iteratively adding complexity with more regulators.

The intuition behind the above steps is the following: The gene sparsification step provides different ways of representing each gene as a function of a small number of regulators. This leads to clusters with high consistency across random train-test sets, since only the most strong dependencies are taken into account in the *K−*means clustering step. The latter is a very simple and fast step, since the vectors being clustered are sparse. The clusters created in this step contain genes whose sparse representation contains the same "most informative" regulators. Then, in the centroid sparsification step, CaMoDi does not use the sparse representation of the genes any more, but reverts to using the actual gene expressions and the "crude" clusters created before, to find a good sparse representation of the centroid of each cluster via cross-validation on the training set. Only the best clusters are kept, and the remaining ones discarded. Then, the sparsity level of the remaining genes is decreased. This step allows for cluster discovery over genes which need more regulators to be correctly clustered together. The reason that CaMoDi starts from very sparse representations is that it searches for the simplest dependencies first and then moves forward iteratively to discover more complicated clusters.

**Figure 1:**
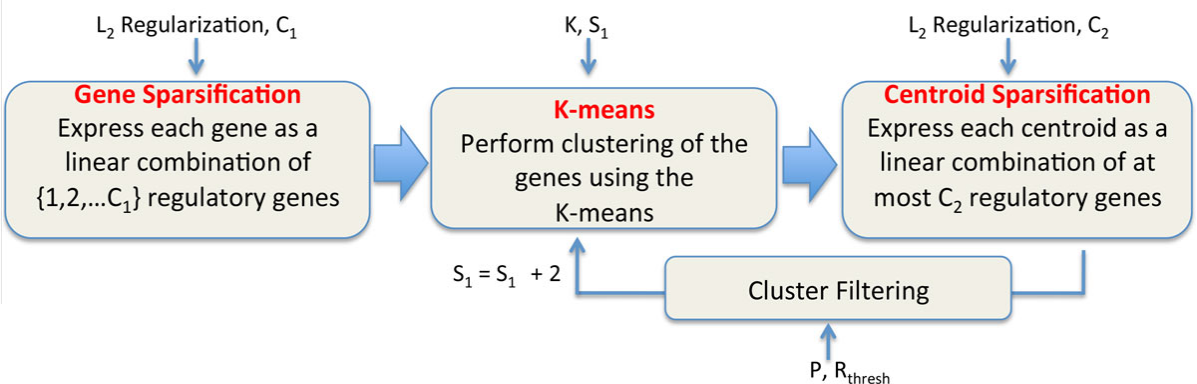
**Graphical representation of CaMoDi's steps**.

Fig 1 presents the flow of the algorithm. There are 6 main parameters which could non-trivially affect the performance of CaMoDi: the two *L*_2_-penalty regularization parameters, the initial sparsity *S*_1 _of the genes, the minimum sparsity of the centroids *C*_2_, *K *in the *K−*means algorithm, and *P *, the percentage of clusters to be retained in each step.

Both CaMoDi and AMARETTO use similar building blocks (e.g., elastic net regularization) in order to discover clusters of genes which are co-expressed using a few regulatory genes. Therefore, we highlight here the main algorithmic differences between the two approaches and the impact of these differences on the expected performance.

CaMoDi clusters the genes based on their sparse representation as a linear combination of regulators. Genes are first mapped to sparse vectors of varying sparsity levels, and then *K−*means clustering is performed on this sparse representation to identify modules. In other words, we combine the genes, not by using their expression across patients, but rather using their sparse projection onto the regulatory gene basis. This leads to a fast implementation that scales well with the number of patients and genes. On the other hand, AMARETTO performs the clustering in a patient-dimension space. This entails significant complexity for AMARETTO when the number of patients associated with the data set is large, as is typical of large data sets such as for Pan-Cancer applications.

In AMARETTO, the iterations continue as long as there exist genes which are more correlated with the centroids of other clusters than with the one they belong to. In CaMoDi, every iteration discards bad clusters, and a new sparse representation of the genes is employed to discover different clusters using the fast *K−*means algorithm. The iterations in CaMoDi explicitly allow for module discovery with different, and in fact increasing, model complexity, which is not the case in AMARETTO. So CaMoDi has the tendency to provide simpler modules, since it explicitly searches for good clusters, which arise from gene sparsification with only a few regulators.

CaMoDi essentially splits the problem of clustering into two subproblems: In the first, it uses the sparse approximations of each gene to create clusters with the *K−*means algorithm. In the second, it finds the best sparse approximation of the centroid of each cluster by using the original expression values. In AMARETTO, both the clustering and the centroid sparsification steps are performed sequentially using the gene expression data until the algorithm converges. Using the initial gene expression data leads to high dependency of the clusters created from the random split of train-test data.

In AMARETTO a gene is re-assigned to the cluster with which it is most positively correlated, whereas in CaMoDi we use the Euclidean distance between the sparse representation of the genes in order to cluster them in the same module.

#### CONEXIC

We now describe **CONEXIC**, introduced by [5]. This will serve as a benchmark for comparing against CaMoDi and AMARETTO in order to demonstrate the properties of each algorithm. CONEXIC is a Bayesian network-based computation algorithm which integrates matched copy number (amplifications and deletions) and gene expression data from tumor samples to identify driver mutations. Inspired by [2], it constructs modules in the form of regression trees based on a Bayesian score-guided search to identify combinations of genes that explain the expression behavior across tumor samples. Particularly, each regression tree contains two building blocks: the *decision nodes *and the *leaf nodes*. A decision node is described by a regulatory gene and a threshold value which specifies how the tree should be traversed. For each tumor sample, one starts from the root node and compares the gene expression of the regulatory genes in each decision node with the corresponding threshold value to move to the right or left child. Each leaf node contains a conditional probability distribution which models the distribution of the expression of the genes of this module that have reached this specific leaf. CONEXIC uses a NormalGamma distribution to model the joint statistics of the genes and the candidate drivers; conditioned on a specific module, the expression of the genes belonging to the module is modeled as a Gaussian distribution. Next we give an overview of the two main steps of CONEXIC.

**Single modulator step: **The goal of this step is to produce an initial clustering of the genes that will serve as input to the next step. Specifically, each gene is associated to the single driver gene that fits it best. Then, a cluster is created by putting together all the genes for which the same driver gene was found to be the best fit. The input to this step is a list of candidate modulators (driver genes), the copy number variation (CNV) data and the gene expression data.

**Network learning step: **This step is based on the Module Networks algorithm by [2,7], and it takes as input the candidate modulator list, the gene expression data and the modules generated by the Single Modulator step. These modules serve as a starting point for the Network learning step, whose goal is to improve the score of the modules and their regulator programs. To do this, the algorithm iterates between learning the regulation program of each module, and re-assigning each gene into the module that best models its behaviour. The re-assignment is based on a scoring function, and the algorithm finishes when the number of re-assignments is below a threshold.

#### Main differences between CaMoDi and CONEXIC

Even though both algorithms aim to discover clusters of genes whose gene expression is driven by a small number of regulators, the approach followed by each of them is significantly different.

First, CONEXIC uses a Bayesian approach to identify the modules, whereas CaMoDi uses linear regression models. In theory, the former could potentially describe more complex dependencies in a data set, but as we observe in this work, this comes at the price of a significantly more complex algorithm, without a commensurate improvement in the quality of the discovered modules. Second, unlike the other two approaches, CONEXIC combines gene expression data and Copy Number Variation (CNV) data to identify modules and their driver genes, whereas CaMoDi (or AMARETTO) only uses the gene expression data. Despite this difference, we show that CaMoDi gets the same and even better performance, with respect to several performance criteria, as compared to CONEXIC with a significant lower run time and algorithmic complexity. Third, CaMoDi's parameters are explicitly related to the important characteristics of the discovered modules, such as the maximum number of regulators in each cluster. Conversely, CONEXIC's parameters only implicitly influence the final clusters, with the performance results being highly dependent on the particular parameter configuration.

### Notation

To argue the merits of the above methods, we need to place the above algorithms on a common platform.

Let's denote by *n *the number of genes and by *p *the number of regulatory genes. Denote a module M={G,R}, where  G and  R are the set of indices of genes and regulators that belong to it, respectively. Finally, we refer to the *m*-dimensional vectors g*_i_, i *∈ 1, . . . , *n*, and $g_{j}^{\left(r\right)}$, *j *∈ 1, . . . , *p*, as the expression of the *i^th ^*gene and the *j^th ^*regulatory gene across *m *samples, respectively, and to the (*n *+ *p*)-dimensional vector **s**^(*k*) ^as the vector expression corresponding to the *k^th ^*patient.

For simplicity of the exposition, fix any module M={G,R} generated by either algorithm. For any given sample **s**^(*k*)^, the module discovery algorithm is trying to predict the value of ${\left\{s_{i}^{\left(k\right)}\right\}}_{i\in G}$, that we denote by ${\left\{{\tilde{\text{s}}}_{i}^{\left(k\right)}\right\}}_{i\in G}$, based on ${\left\{{\text{s}}_{j}^{\left(k\right)}\right\}}_{j\in R}$, i.e., ${\tilde{\text{s}}}_{i}^{\left(k\right)}=f\left({\left\{{\text{s}}_{j}^{\left(k\right)}\right\}}_{j\in R}\right),\forall i\in G$, where the function *f *(·) captures the model considered by a given procedure.

AMARETTO and CaMoDi cluster together genes whose expression is well approximated by a linear combination of the same few regulatory genes, and therefore the module  M is associated with a set of nonnegative coefficients ${\left\{\alpha_{j}\right\}}_{j=1}^{p}$. Thus the *j^th ^*regulator is part of the set  R iff *α_j _≠ *0. Given a new sample **s**^(*k*)^, the predicted value of all the genes in  G is ${\tilde{\text{s}}}_{i}^{\left(k\right)}=\sum_{j\in R}\alpha_{j}{\text{s}}_{{}_{j}}^{\left(k\right)},\forall i\in G$.

CONEXIC does not assume a linear dependency model between regulators and genes. Recall that CONEXIC models each module as a regression tree, where each node is associated with a regulator and a threshold value, and the leafs are associated with a Gaussian distribution of some mean and variance. So, the set of all the different regulatory genes that appear on the decision nodes of the tree constitutes the regulator set  R of this module. Given a new sample **s**^(*k*)^, we traverse the tree until we reach a leaf based on the expression of the regulatory genes of the specific sample. The mean value of the corresponding leaf, denoted as $\mu_{leaf}^{\left(k\right)}$, indicates the expected value of all the genes in **s**^(*k*) ^which belong to the module. Therefore, in CONEXIC, the predicted value of ${\left\{{\text{s}}_{i}^{\left(k\right)}\right\}}_{i\in G}$ based on ${\left\{{\text{s}}_{j}^{\left(k\right)}\right\}}_{j\in R}$ is given by the mean value stored on the leaf reached when **s**^(*k*) ^traverses the tree, i.e.,${\tilde{\text{s}}}_{i}^{\left(k\right)}=\mu_{leaf}^{\left(k\right)},\forall i\in G$.

### Data

We now describe the data upon which we will evaluate the different approaches that were discussed above. As stated in the Introduction, in this work we use the Pan-Cancer data to help uncover underlying genomic patterns in several different tumors and combinations of tumors. Next we describe this data in more detail:

**Gene expression data: **This data is part of the Pan-Cancer initiative provided by The Cancer Genome Atlas (TCGA). It consists of the expression value of 19451 genes for 3452 patients (also referred to as samples) spanning a total of 12 tumor (cancer) types. In our work, we combined the Colon Adenocarcinoma (COAD) with the Rectum Adenocarcinoma (READ) and considered it as one cancer (COAD-READ), since the latter had only 71 samples and it is very similar to the former as far as gene expression is concerned.

**Regulatory genes: **These are a subset of genes which are identified via certain biological regulatory mechanisms and are known to drive other genes. This set has been created based on transcription factor data extracted from the HPRD database [8]. Our data-set consists of 3609 regulatory genes. Note that the set of regulatory genes constitutes a small fraction of the set of all genes.

**Copy Number Variation data (CNV):** Copy Number Variations (CNV) refer to genomic alterations of the DNA of the genome that has been used to implicate genes in cancer growth and progression. CNVs generally correspond to relatively large regions of DNA, usually containing many genes, which have been deleted or duplicated. They often influence the expression of genes in a cluster via changes in the expression of the driver. This data is also part of the Pan-Cancer initiative.

The CNV data is only used by the CONEXIC algorithm, both for the single modulator step and the Network learning step. Note that neither AMARETTO nor CaMoDi make use of this data. However, since we want to use the same data for each of the methods, we only use the gene expression of those genes and patients for which CNV data was available. In this respect, CONEXIC has an explicit advantage in identifying good modules of genes, since it uses more data than the other two approaches.

### Performance and evaluation criteria

In this section, we introduce and explain the performance criteria that will be used in our computational study to test the quality of the modules that each of the methods introduced above discovers. We argue that each of these performance metrics are highly relevant to the problem of identifying statistically significant genomic profiles from given data.

***R squared and adjusted R squared ***(R*^2^, ${\bar{R}}^{2}$): *We use the standard coefficient of determination from statistics to quantify the goodness of approximation of our regression problem. The coefficient of determination, known as *R*^2^, measures how well the expression of the genes which belong to the module are explained by the corresponding regulators. This translates to measuring the Euclidean distance between ${\tilde{s}}_{i}^{\left(k\right)}$ and $s_{i}^{\left(k\right)}$,∀*i *∈ *G*:

$$R^{2}=1-\frac{\sum_{k=1}^{m}\underset{i\in G}{\sum }||{\tilde{\text{s}}}_{i}^{\left(k\right)}-{\text{s}}_{i}^{\left(k\right)}|{|}^{2}}{\sum_{k=1}^{m}\underset{i\in G}{\sum }{∥{\text{s}}_{i}^{\left(k\right)}-\frac{1}{\left|G\right|}\underset{i\in G}{\sum }{\text{s}}_{i}^{\left(k\right)}∥}^{2}},$$

where *m *is the number of samples for which we are trying to make a prediction. High *R*^2 ^means that the residual energy that is not explained by the assigned regulatory genes is relatively small, so we are interested in clusters with high *R*^2 ^values. In order to adjust for the number of regulators relative to the number of patients (samples), we compute the adjusted *R*^2 ^for each module  M, defined as

$${\bar{R}}^{2}\overset{{}^{\circ}}{=}R^{2}-\left(1-R^{2}\right)\frac{\left|R\right|}{m-|R|-1}.$$

**Consistency **(*S*): When evaluating the performance of an algorithm, it is important to quantify how consistent the algorithm is with respect to the selection of the training set. Specifically, given a gene expression dataset, a typical way of testing the consistency of the model generated by a statistical learning algorithm is to randomly use 70% of the samples for training and 30% for testing. Ideally, we would like the algorithm to generate similar modules independent of the random split of the data. A standard measure used for comparing the similarity of two sets is the Jaccard index. The Jaccard index between two sets  A and  B is given by

$$J\left(A,B\right)=\frac{\left|A\cap B\right|}{\left|A\cup B\right|},$$

i.e., by the ratio of cardinalities of the intersection and the union between the two sets. In our case, the two sets *A *and *B *will consist of the sets of genes (or regulators) that belong to two different modules. Denote two different modules as $M_{\text{1}}=\left\{G_{1},R_{1}\right\}$ and $M_{\text{2}}=\left\{G_{2},R_{2}\right\}$. We define the average Jaccard index between $M_{\text{1}}$ and $M_{\text{2}}$ as

$$\bar{J}\left(M_{1},M_{2}\right)=\frac{J\left(G_{1},G_{2}\right)+J\left(R_{1},R_{2}\right)}{2}.$$

To demonstrate how the consistency measure *S *is calculated, assume we run the algorithm two times (2 random bootstraps), and each run produces *N_a _*and *N_b _*modules, respectively, denoted as ${\left\{M_{i}^{a}\right\}}_{i=1}^{N_{a}}$ and ${\left\{M_{i}^{b}\right\}}_{i=1}^{N_{b}}$. Then, for every module $M_{i}^{a}$, find the $M_{j}^{b}$ module which maximizes $\bar{J}\left(M_{i}^{a}M_{j}^{b}\right)$, i.e., the module in the second run to which it is most similar to on the average. Perform the same procedure after interchanging the role of the first and the second bootstrap. These steps lead to *N_a _*+ *N_b _*pairs of modules, each one of which has a corresponding average Jaccard index. Averaging over all the pairs computes the consistency *S*. In the general case of *B *bootstraps, we will repeat the above procedure for every pair of bootstraps, and compute the final robustness *S *as the average of the robustness of each pair of bootstraps.

**Homogeneity **(*H*): This performance metric captures whether the genes inside a module demonstrate fairly correlated expression with each other or not. We use the standard Pearson Correlation coefficient for this purpose, denoted by *ρ*. Specifically, given a module and the set of genes that belong to it, we compute for each gene the correlation coefficient of that gene with all the rest. Thus for each gene we have a *ρ *value. The homogeneity of that module is given by the average of all the computed coefficients across all the genes that belong to the module. Finally, the homogeneity metric *H *is computed as the average of the homogeneity values across the clusters.

### Simulations

#### Preprocessing of the data

Before making use of the data, we normalize each gene expression vector to have zero mean and standard deviation one. We make sure that all the samples that we use have both gene expression data and CNV data. Finally, we apply a variance filter to the training set to retain only the 15% most varying genes (approximately 3000 genes). The reason for choosing only 15% of the genes is mainly due to the high run time of CONEXIC and AMARETTO for larger datasets. We use 70% of the samples as training and evaluate the results on the remaining 30% of the samples.

#### Parameter selection

When comparing different algorithms, choosing the correct parameter configuration is an important issue, especially when it comes to methods which have many parameters which could potentially affect the results. We perform an optimization procedure to identify the parameter configuration for CONEXIC, CaMoDi and AMARETTO: starting from a predefined parameter configuration, in each iteration, we identify the parameter which leads to the highest increase in the average ${\bar{R}}^{2}$ performance, until no parameter could lead to a significantly better performance. We then use that configuration for all the simulations performed in this work. For a detailed description of our optimization procedure see the Additional File 1.

#### Machine specifications

The machine used to conduct the experiments has the following specifications: 16 GB RAM, Inter Core i5-4430 CPU at 3.00GHz *× *4 and Ubuntu 13.14. AMARETTO and CaMoDi are written in Matlab, and CONEXIC in Java. However, all the methods are called from Matlab (version R2011a). The run time of each method is captured while running 4 Matlab sessions concurrently, each session running all the methods for a specific dataset. Spasm Toolbox [9], a Matlab toolbox for sparse statistical learning, is employed for the elastic-net regression in both AMARETTO and CaMoDi.

## Results

To analyze the different performance of the three methods and understand the strengths and weakness of each of them, it is important to perform exhaustive simulations with datasets of different characteristics. With this in mind, in this work we present several simulation results, which can be divided into three groups. First, we compare the performance of all the methods across all the individual tumor datasets (11 in total), and compare the modules found by each of the methods. Then, we consider combinations of the individual tumors, in an attempt to discover intertumor modules. We present the results for eight different tumor combinations across the three methods. Finally, we employ CaMoDi across all tumors on the complete TCGA Pan-Cancer dataset, to demonstrate its capabilities as a scalable cancer module discovery algorithm.

**Figure 2:**
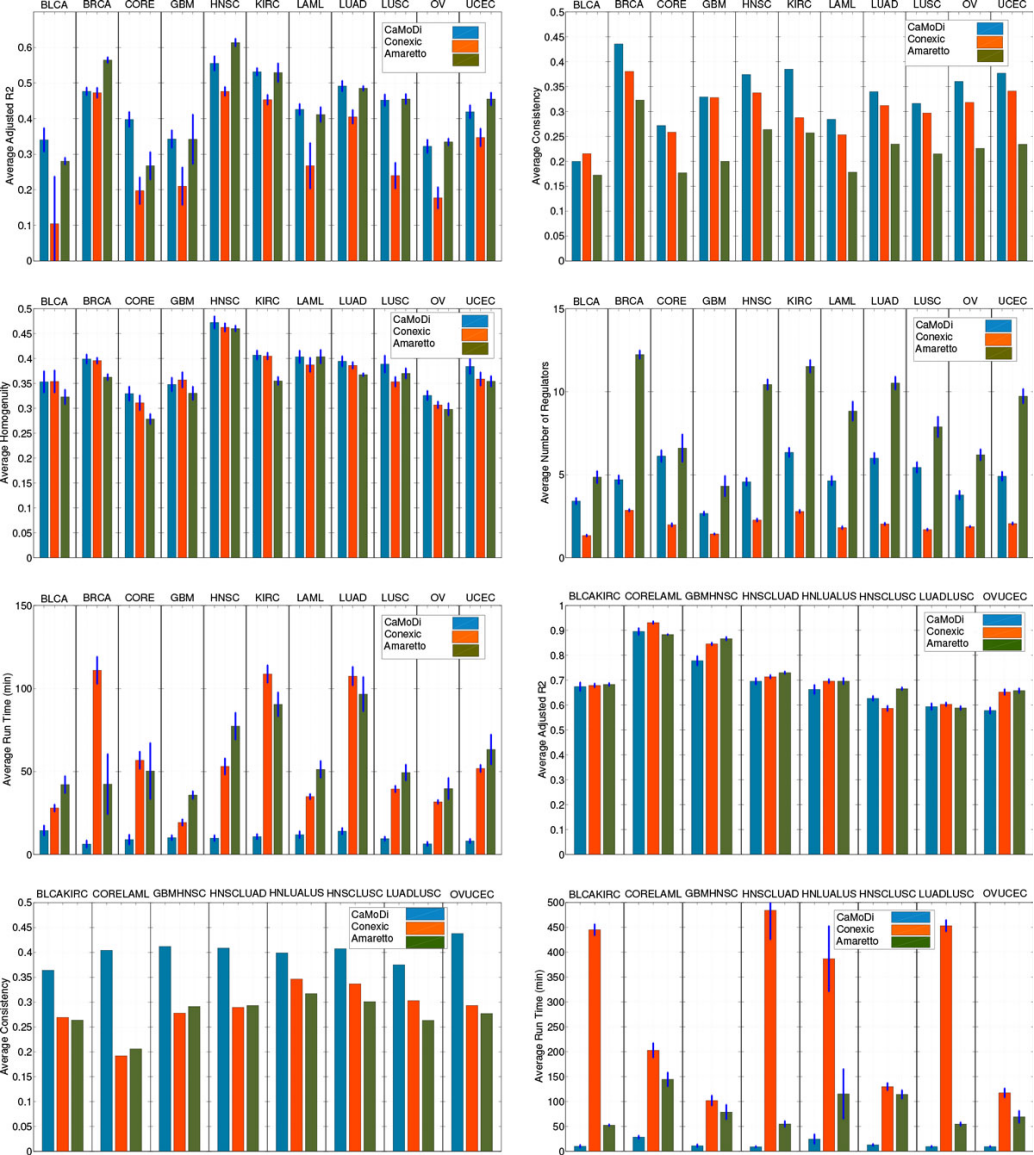
**Performance comparison**. *CORE *stands for *COADREAD*, and *HNLUALUS *for *HNLUADLUSC*.

**Individual tumors**: For the individual tumors we run each method using the samples of each tumor separately. Specifically, to make the comparison fair across all methods, for every bootstrap (a random 70 *- *30 train-test split), we perform the following procedure: we retain the clusters that contain between 5 and 1000 genes, and sort them based on their ${\bar{R}}^{2}$. We filter the best clusters which cover at least 80% of the genes and average all the results over these clusters. The choice of 80% is not crucial and similar results are obtained for different choices, as shown in the Additional File 1. Furthermore, note that the purpose of the three approaches is not to create a module network which explains all the genes, but to identify a set of good modules which contain co-expressed genes (a similar argument is made in [5]). For example, in CONEXIC it is possible to create "garbage" modules containing the "bad" clusters. The results are summarized in Fig.2. Specifically, we show for each method and tumor the average ${\bar{R}}^{2}$, Consistency *S*, homogeneity *H*, run time and number of regulators per module. The remaining results are collated in the Additional File 1.

**Combination of tumors**: With this set of simulations we address the problem of module identification across tumors. In this case, for every bootstrap (10 in total), we combine 70% of the data of the tumors under consideration in the train set and leave the remaining 30% in the test set. Then, we perform the preprocessing steps described in Section. Finally, the methods treat each sample in the same way to construct modules of genes that are agnostic to the tumor knowledge. Fig.2 presents the results for: *BLCA-KIRC, COADREAD-LAML, GMBHNSC, HNSC-LUAD, HNSC-LUAD-LUSC, HNSC-LUSC, LUAD-LUSC *and *OV-UCEC*. Due to space limitations, we only show the results related to the average ${\bar{R}}^{2}$, Consistency *S *and run time, and refer the reader to the Additional File 1 for the remaining metrics.

**Pan-Cancer dataset: CaMoDi performance**: For completeness, and to show the potential of CaMoDi when applied to large datasets, we perform one last simulation that combines together the data of all the tumors presented in the Pan-Cancer dataset. We combine the samples in the same way as for the combination of tumors. However, in this case we only present the results for CaMoDi, since CONEXIC required prohibitively long times (more than 48 hours of run time for each bootstrap as compared to less than 1.5 hours for CaMoDi). Due to space limitations, these results are shown in the Additional File 1.

## Discussion

The performance results from the individual tumor experiments (Fig. 2) demonstrate that CaMoDi outperforms CONEXIC and AMARETTO in the average homogeneity and consistency metrics across all the individual tumors except in the *GBM *data for the homogeneity and the *BLCA *data for the consistency (7 out of 8 different datasets). This demonstrates the robustness and consistency of CaMoDi with respect to the random train-test split of the data. Regarding the average ${\bar{R}}^{2}$, we observe that CaMoDi outperforms CONEXIC in all cases, with CaMoDi and AMARETTO achieving comparable average ${\bar{R}}^{2}$ values. Specifically, CaMoDi outperforms AMARETTO in 4 out of the 11 cases, in 4 other datasets it gets lower average ${\bar{R}}^{2}$, and in the remaining 3 datasets the performance of the two algorithms is comparable. One of the main strengths of CaMoDi is its low run time. Specifically, we observe that the proposed algorithm runs in approximately the same time (less than 10 minutes) for all the individual tumors, achieving an order of magnitude improvement (10 times faster against CONEXIC) over the other two algorithms. We observe that AMARETTO tends to employ a high number of regulators per module (more than 9 regulators in 5 out of the 11 individual tumors), whereas CONEXIC uses less than 4 regulators per module on average in all the individual tumors. CaMoDi finds a good balance between these two methods, with less than 5 regulators on average in 7 out of the 11 datasets, and less than 7 in the remaining ones. This implies that CaMoDi is able to obtain good performance with a lower average module complexity, a feature also demonstrated by CONEXIC. We note that CaMoDi discovers novel modules that are also unique compared to the other two methods. A statistical comparison of the Jaccard index between the discovered modules of CaMoDi and the remaining two algorithms in three datasets is presented in the Additional File 1. In short, we observe that more than 30% of the discovered clusters of CaMoDi have a maximum Jaccard index of 0.1 with any cluster of CONEXIC and AMARETTO, i.e., a relative high percentage of clusters have very few genes in common with any cluster from the other two methods.

The results for the combined tumor experiments (Fig. 2) demonstrate that CaMoDi still outperforms CONEXIC and AMARETTO with respect to the consistency metric in all the combinations, while achieving a comparable performance with respect to the homogeneity metric (cf. Additional File 1 ). In terms of average ${\bar{R}}^{2}$, we observe similar results for the three algorithms. Yet, the run time of CaMoDi averages 15 *− *20 minutes, whereas that of CONEXIC and AMARETTO increases significantly with respect to the individual tumors. This is especially noticeable for the case of CONEXIC, where some datasets needed as long as 6 hours to generate the module network for one bootstrap. These results reinforce that CaMoDi is an efficient algorithm which discovers high quality modules even in tumor combinations, while requiring an order of magnitude less time to run than CONEXIC and AMARETTO. Further, even in the case of combinations, CaMoDi provides modules with significantly lower average number of regulators than that of AMARETTO (cf. Additional File 1 ).

We additionally demonstrate the capabilities of CaMoDi by employing it for the entire Pan-Cancer dataset. These results appear only in the Additional File 1 where we observe that CaMoDi was able to discover 30 modules that cover 15% of all the genes with an average ${\bar{R}}^{2}$ of 0.7, while keeping an average number of 7 regulators per cluster.

To summarize the numerical findings, we have demonstrated that CaMoDi is an algorithm that produces modules of high quality, while requiring significantly less run time than CONEXIC and AMARETTO. We note that the choice of using 15% of the genes for the simulations was restricted by the computational complexity limitations of CONEXIC, not by CaMoDi. In addition, the performance of CONEXIC requires the CNV information to acquire the initial modules, which is not the case for CaMoDi or AMARETTO. Finally, it should be highlighted that CaMoDi has six easily interpretable parameters which affect its performance, the values of which can be optimized using a cross-validation method for each dataset separately. Due to the large number of parameters and the long run time for CONEXIC and AMARETTO, this performance optimization step was not employed in our experiments. Finally, we remark that a detailed study of the biological implications of cancer modules discovered by CaMoDi is an ongoing research endeavor, which we reserve for future studies.

## Conclusions

In this paper we present CaMoDi, a novel method for Cancer Module Discovery. We demonstrate through extensive simulations on the TCGA Pan-Cancer dataset that CaMoDi achieves comparable or better performance than that of CONEXIC and AMARETTO on several performance metrics, while demanding on average an order of magnitude less computation time. Moreover, CaMoDi is an algorithm that scales very well with the number of genes and the number of samples, making it suitable for module discovery in large datasets. We demonstrate the performance with respect to several important metrics, such as *R*^2^, adjusted *R*^2^, homogeneity and consistency, and run time. Finally, CaMoDi is an algorithm with a few intuitive parameters controlling its performance. This is especially important in the current era of increasing volumes of datasets on which we need to perform complicated tasks of biological inference.

## Abbreviations

CNV: Copy Number Variation; TCGA: The Cancer Genome Atlas; CORE: COAD and READ cancers; HNLUALUS: HNSC, LUAD and LUSC cancers.

## Competing interests

The authors declare that they have no competing interests.

## Authors' contributions

AM, IO, KV and OG conceived of the project idea. AM and IO implemented the CaMoDi algorithm in Matlab, OG implemented the AMARETTO algorithm in Matlab. AM, IO designed the experiments. AM, IO, KV performed the simulations. All authors contributed to the analysis and interpretation of the results. AM, IC, KV wrote the first version of the manuscript, and AM, IO, KV, AG and OG were involved in revising it. All authors read and approved the final manuscript.

## Software implementation

CaMoDi is written in Matlab, and it is available at: http://web.stanford.edu/~amanolak/CaMoDi.html. We also provide the necessary files and code to perform all the simulations presented in the paper.

## Supplementary Material

Additional File 1 **It provides detailed information on the parameter optimization procedure used to identify the parameter configuration for CONEXIC, CaMoDi and AMARETTO, and it also presents additional simulation results, e**.g., the CaMoDi performance on the Pan-Cancer dataset. The format is .PDF, and it is available at BMC Genomics online. Click here for file

## Declarations

The publication costs for this article were funded by the NSF Center for Science of Information (CSoI) grant agreement CCF-0939370.

This article has been published as part of *BMC Genomics *Volume 15 Supplement 10, 2014: Proceedings of the 25th International Conference on Genome Informatics (GIW/ISCB-Asia): Genomics. The full contents of the supplement are available online at http://www.biomedcentral.com/bmcgenomics/supplements/15/S10.
