# Supplementary material for: CaMoDi: a new method for cancer module discovery
Source: BMC Genomics. 2014 Dec 12;15(Suppl 10):S8. doi: 10.1186/1471-2164-15-S10-S8 (PMC4304219; doi:10.1186/1471-2164-15-S10-S8)
Supplement: Additional File 1 — It provides detailed information on the parameter optimization procedure used to identify the parameter configuration for CONEXIC, CaMoDi and AMARETTO, and it also presents additional simulation results, e.g., the CaMoDi performance on the Pan-Cancer dataset. The format is .PDF, and it is available at BMC Genomics online. [file 1471-2164-15-S10-S8-S1.pdf]

Additional File 1

I. Parameter optimization for CaMoDi, CONEXIC and AMARETTO

We perform the following parameter optimization procedure to identify the parameter configuration for CONEXIC, CaMoDi and AMARETTO. For each method, we fix all the parameters to an initial value (e.g., the initial configuration for CONEXIC is the one shown in [Akavia *et al.*, 2010]), optimize sequentially each parameter by running 10 bootstraps of a $70-30$ split of the GBM data for 5 different values of the current parameter. The specific values used for each parameter can be found in the scripts ConexicOptimization.m, CaMoDiOptimization.m, AmaretoOptimization.m. Then, we choose the value of the parameter which leads to the maximum increase in average $\bar{R}^2$ only if the latter is at least 5% better than the previous configuration. Note that we do not allow self regulation inside a module; i.e., a gene can not belong simultaneously to the set of genes and the set of regulators of a cluster.

The parameters that we optimize over in CONEXIC can be found in the extended manual of CONEXIC [Manual Conexic], from where we chose the 8 parameters which appear to influence the results the most. The parameter optimization of CaMoDi and AMARETTO is much simpler, since for each method there are essentially 6 and 3 parameters, respectively, which can influence the performance. We present the final configuration used for all the simulations presented in this work in Table I.

II. Additional simulation results

A. Individual Tumors

In this section, we add the performance metrics of R^2, and average number of clusters generated by each method for the individual tumor experiment described in the main document in Fig. 1. We observe that average R^2 has a very similar behavior as the average $\bar{R}^2$.

B. Combination of tumors

We show the homogeneity results (Fig. 2-(a)) for all the combinations of tumors presented in the manuscript, as well as the average number of regulators (Fig. 2-(b)) used by each of the methods.

CaMoDi		CONEXIC		AMARETTO	
Parameters	Value	Parameters	Value	Parameters	Value
L2 Gene Sparcification	2	Alpha	2.5	Stop	-20
L2 Centroid Sparcification	1	Lambda	1.5	Percentage Genes Stop	0.05
Min. Sparse Level Centroids	-20	Num. Leafs Penalty	30	Num. Clusters	50
K (k-means)	40	Stop Threshold	0.05		
P (percentage to keep)	10	Min. Cluster Size	20		
		Max. Reassignment Steps	3		
		Num. Regulator Penalty	30		
		Num. Leaf Maximum	6		

TABLE I
Parameters that we optimized over in CaMoDi, CONEXIC and AMARETTO, and the value used for each of them in all the simulations presented in this work.

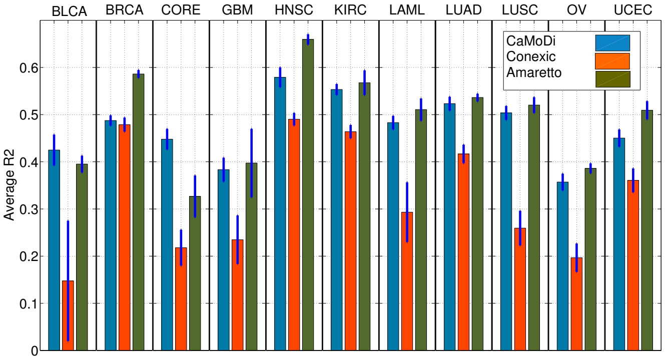

(a) Average R^2

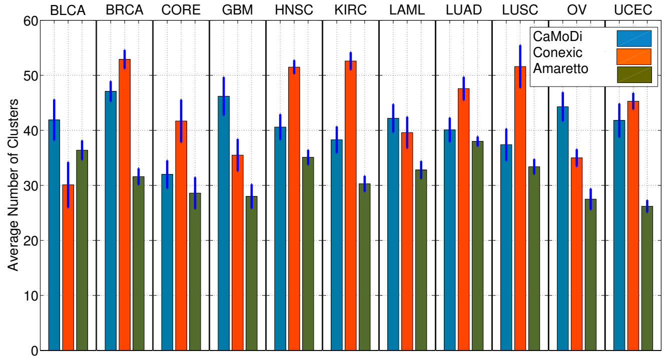

(b) Average number of clusters

Fig. 1. Individual tumor experiments.

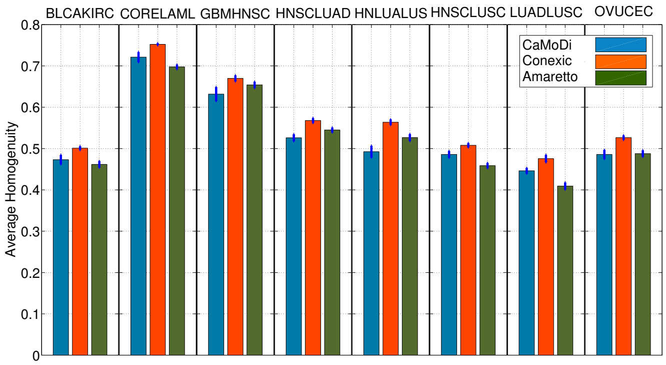

(a) Homogeneity

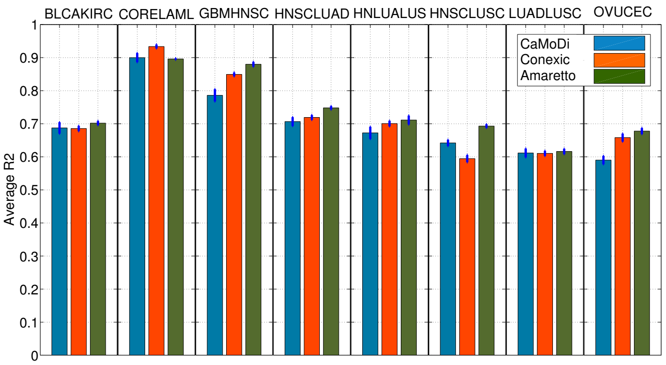

(b) Average R^2

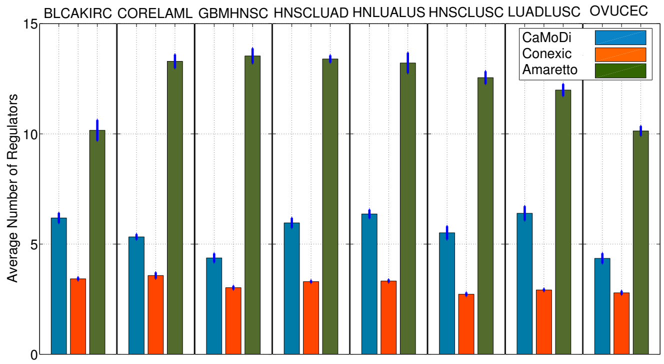

(c) Average number of regulators

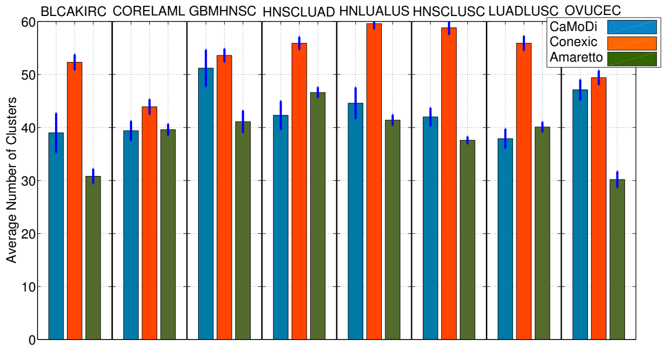

(d) Average number of clusters

Fig. 2. Combination tumor experiments.

C. Average performance results over clusters containing 25% of the genes

In this section, we show the same results as those appeared in Fig. 2 of the main document, but averaged over only the best clusters which contain 25% of the genes (in the main document, the results are averaged over the clusters which contain 80% of the genes). This leads to approximately 600 genes. Results are shown in Fig. 3. We observe that averaging over only a few very good clusters, still leads to the same comparative conclusions as those presented in the main document.

D. Pan-Cancer dataset: CaMoDi performance

In Fig. 4 we present the performance results of CaMoDi when we combine 70% of the samples of each of the 11 individual tumors and test the results with the remaining 30% of the samples. As we already

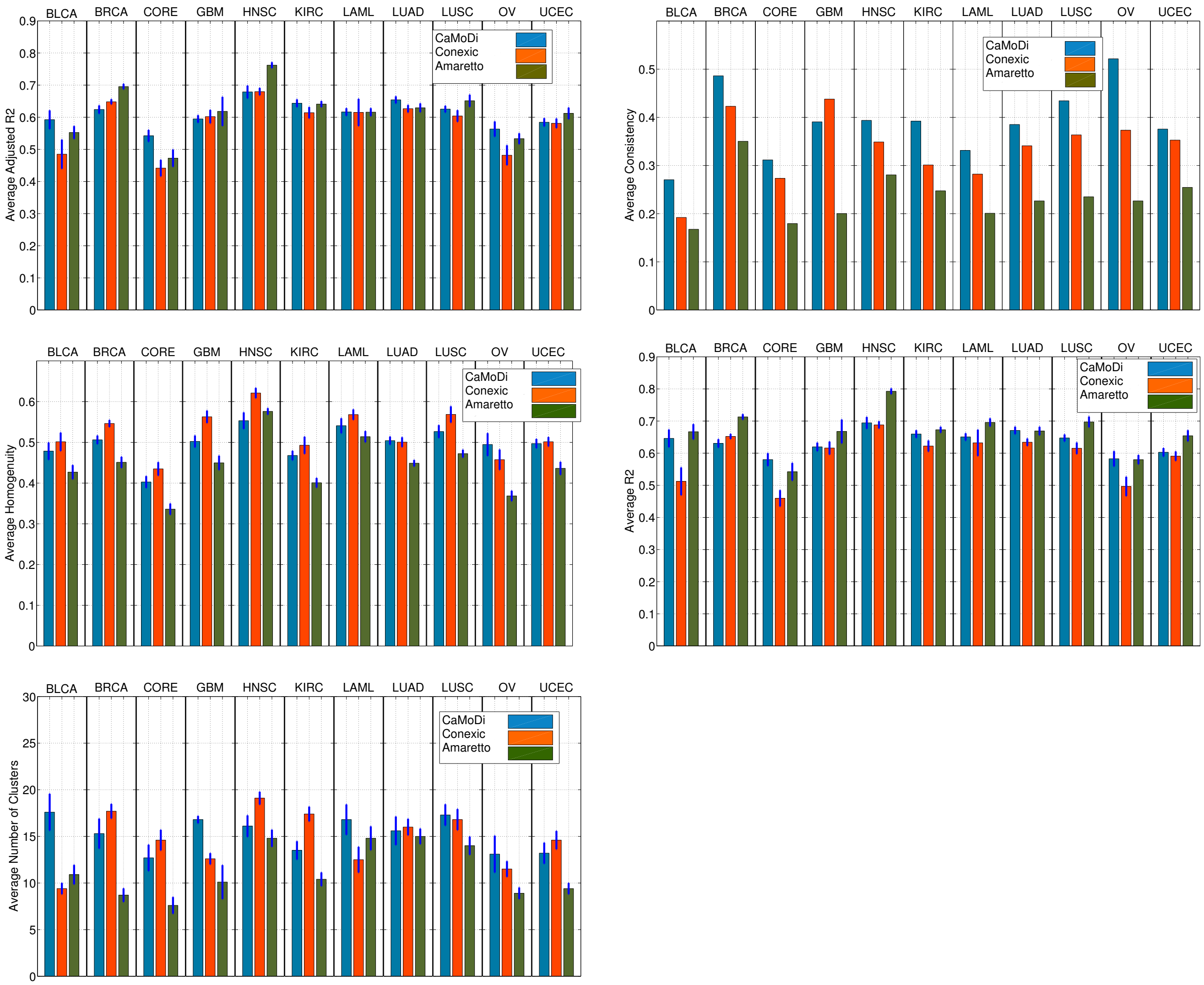

Fig. 3. Performance comparison of CaMoDi, CONEXIC, AMARETTO for the single tumors taking.

described in the main document, it was practically impossible to run the remaining two methods due to extremely high running times.

E. Module Jaccard similarity comparison

In Fig. 5 we show for three different datasets (OV, GBM, HNSC), the histogram of the Jaccard index of CaMoDi's modules with the other two methods. Fix any random bootstrap, for every module generated with CaMoDi, we identify the module of AMARETTO (CONEXIC) with which it has the highest Jaccard index (i.e., it is most similar with) and plot the resulting average histograms across 10 bootstraps. A small Jaccard index means that the corresponding modules are significantly different, whereas a high would suggest the two modules have several genes in common. Observe that in all three datasets, there exist more than 30% of modules discovered with CaMoDi that have a Jaccard index less than 10%, which means that there are many new modules in CaMoDi which are not discovered by AMARETTO or CONEXIC. The biological implications of these modules remains to be examined in future work.

References

[Akavia *et al.*, 2010] Akavia, Uri David, et al. "An integrated approach to uncover drivers of cancer." Cell 143.6 (2010): 1005-1017.

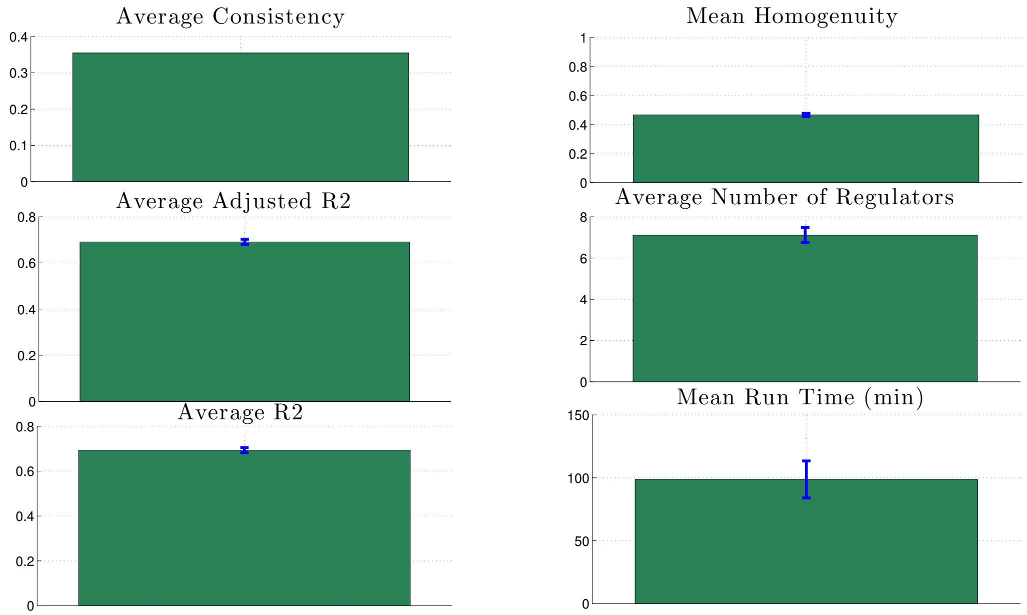

Fig. 4. Pan-Cancer dataset: CaMoDi performance.

[Manual Conexic] http://www.c2b2.columbia.edu/danapeerlab/html/CONEXIC/CONEXICTutorial.pdf

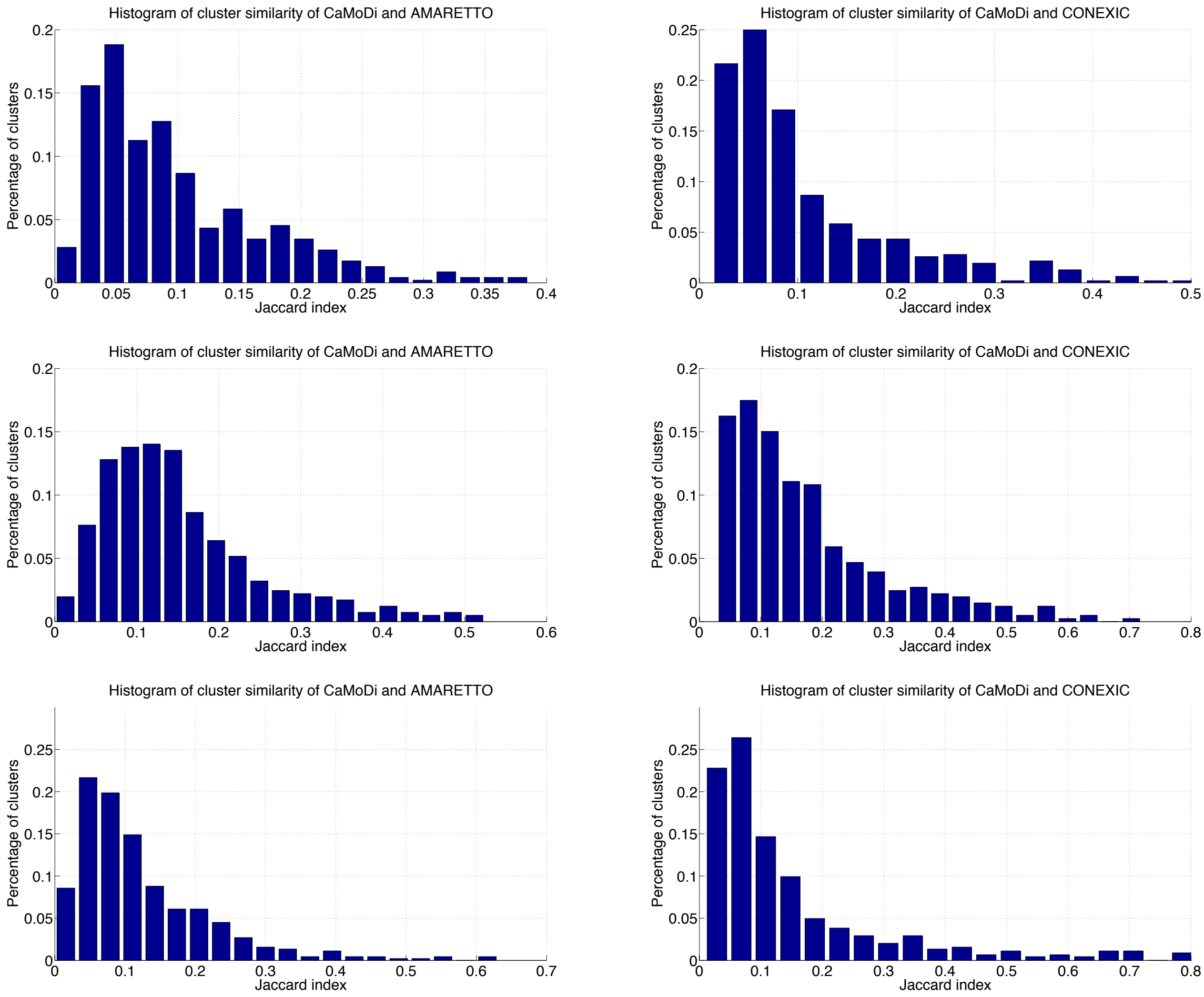

Fig. 5. Module Jaccard similarity comparison of CaMoDi with AMARETTO and CONEXIC for three datasets: GBM, HNSC, OV.
